# SGLT-2 Inhibitors’ and GLP-1 Receptor Agonists’ Influence on Neuronal and Glial Damage in Experimental Stroke

**DOI:** 10.3390/biomedicines12122797

**Published:** 2024-12-10

**Authors:** Anna Murasheva, Oksana Fuks, Natalya Timkina, Arina Mikhailova, Timur Vlasov, Konstantin Samochernykh, Tatiana Karonova

**Affiliations:** Almazov National Medical Research Centre, 197341 Saint-Petersburg, Russia; fuks_os@mail.ru (O.F.); n.timkina2014@yandex.ru (N.T.); armikhaylova@yandex.ru (A.M.); tvlasov@yandex.ru (T.V.); samochernykh_ka@almazovcentre.ru (K.S.); karonova@mail.ru (T.K.)

**Keywords:** stroke, diabetes mellitus, neuroprotection, neuronal damage, SGLT-2 inhibitors, GLP-1 receptor agonists

## Abstract

**Background:** SGLT-2 inhibitors (SGLT-2i) and GLP-1 receptor agonists (GLP-1RA) have demonstrated nephro- and cardioprotective effects, but their neuroprotective properties, especially concerning stroke severity, and mechanisms are not unambiguous. We aimed to study the influence of SGLT-2i with different selectivity and GLP-1RA on brain damage volume and neurological status in non-diabetic and diabetic rats and to investigate the underlying mechanisms. **Methods:** Non-diabetic *Wistar* rats were divided into five groups (n = 10 each) and received empagliflozin, canagliflozin, or dulaglutide as study drugs and metformin as comparison drug. Control animals were administered 0.9% NaCl for 7 days before stroke. At 48 h after stroke, we assessed neurological deficit, neuronal and astroglial damage markers, and brain damage volume. We also modeled type 2 DM in *Wistar* rats using the high-fat diet+nicotinamide/streptozotocin method and established similar treatment groups. After 8 weeks, rats were subjected to stroke with further neurological deficit, neuroglial damage markers, and brain necrosis volume measurement. **Results**: In non-diabetic rats, all the drugs showed an infarct-limiting effect; SGLT-2i and dulaglutide were more effective than metformin. DULA improved neurological status compared with MET and SGLT-2i treatment. All the drugs decreased neurofilament light chains (NLCs) level and neuronal damage markers, but none of them decreased the glial damage marker S100BB. In DM, similarly, all the drugs had infarct-limiting effects. Neurological deficit was most pronounced in the untreated diabetic rats and was reduced by all study drugs. All the drugs reduced NLC level; dulaglutide and empagliflozin, but not canagliflozin, also decreased S100BB. None of the drugs affected neuron-specific enolase. **Conclusions**: SGLT-2i and GLP-1RA are neuroprotective in experimental stroke. GLP-1RA might be more effective than SGLT-2i as in non-diabetic conditions it influences both brain damage volume and neurological status. All study drugs decrease neuronal damage, while GLP-1RA and highly selective SGLT-2i EMPA, but not low-selective CANA, also have an impact on neuroglia in diabetic conditions.

## 1. Introduction

Ischemic stroke is one of the leading causes of disability and death worldwide. Stroke in general accounts for up to 11.6% of all deaths today, while the ischemic type is the most common, accounting for more than 60% of all strokes [1]. Every year, more than 16% of all strokes occur in people aged 15–49 years, and over 62% in older individuals [2]. Diabetes mellitus (DM), as well as atherosclerosis, hypertension, and other modifiable and non-modifiable parameters, comprise independent risk factors for stroke. Thus, stroke incidence in diabetic patients is at least twice as large as in the general population, while the course and outcomes of stroke are much more severe [3]. Nevertheless, healthcare still lacks effective medicament prophylaxis for stroke and ways to reduce its severity and improve the course and outcome.

In recent years, modern glucose-lowering drugs used for type 2 DM treatment have demonstrated outstanding pleotropic effects, including cardio- and nephroprotective ones, so recently, the type 2 DM treatment paradigm has shifted from a glucose-lowering to an organ-protective one. Two glucose-lowering classes that are at the forefront due to their pronounced protective properties are sodium-glucose cotransporter type 2 inhibitors (SGLT-2is) and glucagon-like peptide-1 receptor agonists (GLP-1RAs) [4]. Both have the potential to reduce the risk of myocardial infarction as well as all-cause and cardiovascular mortality and to realize nephroprotective potential in diabetic patients [5]. However, the data on the neuroprotective potential of GLP-1RA and SGLT-2i are inconclusive. Only two long-acting GLP-1RAs, dulaglutide (DULA) and injectable semaglutide, have been shown to reduce the risk of stroke in people with DM [6]. Although SGLT-2is have not been shown to be neuroprotective against stroke in general, this class of drugs seems to have good prospects, as they may reduce the risk of cardioembolic stroke by influencing the course of atrial fibrillation [7].

Importantly, the results of recently completed randomized control trials (RCTs) demonstrate the ability of SGLT-2i to improve the course of heart failure regardless of the presence of diabetes [8,9]. Thus, the perspective of using modern glucose-lowering drugs for the treatment and prevention of cardiovascular diseases is now one of the most progressive.

The above considerations led us to perform an experimental study of neuroprotective properties of SGLT-2i in comparison with GLP-1RA in terms of influence on the course of stroke and post-stroke recovery in both non-diabetic and diabetic conditions, as well as to investigate the mechanisms of glucose-lowering drugs’ neuroprotective effects in both non-diabetic and diabetic conditions.

The aim was to study the influence of SGLT-2i with different selectivity (empagliflozin and canagliflozin) and long-acting GLP-1RA on brain damage volume and neurological status in both non-diabetic and diabetic rats and to investigate the underlying mechanisms.

## 2. Materials and Methods

### 2.1. Study Design

The study consisted of two parts: the 1st part was conducted in non-diabetic animals and the 2nd part in diabetic ones.

#### 2.1.1. I Part: Stroke Modeling in Non-Diabetic Animals

Male *Wistar* rats weighing 220–250 g underwent 14 days of acclimatization, during which their behavior, skin, visible mucous membrane color, tail position, and body weight gain were assessed. Animals without abnormalities in the above-mentioned parameters were included in the experiment.

Five groups were formed (n = 10 each):

- “Control”, rats receiving 0.9% NaCl once daily per os for 7 days;
- “MET”—metformin 200 mg/kg once daily per os for 7 days;
- “EMPA”—empagliflozin 2 mg/kg once daily per os 7 days;
- “CANA”—canagliflozin 25 mg/kg once daily per os 7 days;
- “DULA”—dulaglutide 0.12 mg/kg every 72 h s.c. for 7 days (3 injections in total).

The choice of doses and regimens of drug administration was based mostly on our previous studies where we demonstrated the high effectiveness of these protocols in the protection of the brain in stroke conditions [10]. Briefly, the doses were determined according to the pharmacokinetics of the study drugs in rats, taking into account existing literature where partly similar experimental protocols were used. Thus, for MET, a dose of 200 mg/kg was shown to be effective in the model of cerebral ischemia–reperfusion injury [11]. The frequency of DULA administration was based on its pharmacokinetics [12], while the dosage was chosen by conversion from the human dosage calculated by body weight, taking into account the accelerated metabolism in rats. As there was lack of information concerning prolonged use of EMPA and CANA before stroke modeling in rats, we also mostly focused on the human dosage per body weight, taking into account the accelerated metabolism in rats.

After 7 days of respective treatment, rats of all groups underwent middle cerebral artery occlusion (MCAO) using the method described by Koizumi [13]. We widely use this method in our experiments, and we have described it in detail before [14,15]. Briefly, anesthetized rats (Zoletil + Xylazine i.m.) were subjected to 30 min of filament transient MCAO. A 20–22 mm long thread (Doccol Corporation, Sharon, MA, USA) was inserted into the internal carotid artery and moved to the middle cerebral artery (MCA) mouth closing it. The effectiveness of the occlusion was verified by the Doppler method (Minimax Doppler-K model NB, Saint-Petersburg, Russia). We utilized the trepanation window to reach the MCA cortical branch. The decrease in linear blood flow by 70% and more after thread insertion allowed us to verify ischemia effectiveness. The 30 min ischemia period was followed by 48 h of reperfusion.

Throughout the anesthesia period, animals’ rectal temperature was maintained stable at the level of 37.0 °C using a thermostatic table. All animals received an i.p. injection of 1 mL 5% glucose solution at the end of the operation, as well as 4–5 h after the start of the reperfusion and thereafter in the reperfusion period if needed.

Next, 48 h after the start of reperfusion period, neurological status was evaluated in all the groups according to the Garcia scale, which is used to measure post-stroke changes and includes sensory and motor parameters. According to it, the most prominent neurological deficit was characterized by 3 points, while the absence of neurological impairment was described as 18 points [16]. After neurological evaluation, all the animals were anesthetized, and blood samples from the caudal vein were collected for further measurement of neuronal and glial damage markers. Blood samples were then centrifugated and serum was aliquoted and frozen. We measured the neurofilament light chain (NLC) level (Enzyme-linked Immunosorbent Assay Kit for Neurofilament, Light Polypeptide (NEFL), Cloud-Clone Corp., Katy, TX, USA) and neuron-specific enolase (NSE) (Enzyme-linked Immunosorbent Assay Kit For NSE, Cloud-Clone Corp., Katy, TX, USA) as a neuronal damage markers and the S100BB (Enzyme-linked Immunosorbent Assay Kit For S100 Calcium Binding Protein (S100), Cloud-Clone Corp., Katy, TX, USA) concentration as a glial damage marker.

Then, all the animals were euthanized, the brains were extracted, and 5 brain slices, each 2 mm thick, were incubated for 15 min in 1% triphenyltetrazolium chloride solution (MP Biomedicals, Santa Ana, CA, USA) at a temperature of 37 °C with pH 7.4. After that, we photographed both sides with the help of an Olympus C4000 digital camera connected with an MBS10 microscope (BioLab, Moscow, Russian Federation). The images were analyzed using ImageJ version 1.54 j (https://imagej.net/ij/ (accessed on 15 August 2024)) and Adobe Photoshop 8.0 software (San Jose, CA, USA) in order to measure the affected zone in per cent from the total volume of the brain.

Glycemia was measured by tail vein puncture with the help of an AccuCheck Performa glucometer (Roche, Basel, Switzerland) in all animals three times during the treatment period, three times during MCAO modeling, as well as in the reperfusion period—no less than three times daily. No measurements were taken after fasting.

#### 2.1.2. II Part: Stroke Modeling in Diabetic Animals

Male *Wistar* rats were subjected to DM modeling as described previously [14,15]. Rats were maintained on a high-fat diet (22% of saturated fats) during the entire experiment. After 4 weeks from the beginning of the study, rats were subjected to an i.p. injection of 230 mg/kg nicotinamide (Sigma-Aldrich, Milwaukee, WI, USA) solution, followed by an i.p. injection of 60 mg/kg streptozotocin (Sigma-Aldrich, Milwaukee, WI, USA) solution. On the 2nd and 3rd days, glycemia was measured by tail vein puncture using the AccuCheck Performa glucometer (Roche, Basel, Switzerland). Rats whose glycemia reached 11.1 mmol/L in two different measurements were diagnosed with DM, while rats whose glycemic level did not reach this value twice were subjected to an oral glucose tolerance test (OGTT). We evaluated glycemia fasting 15, 30, 60, and 90 min after gastric tube gavage of 3 g/kg 40% glucose solution. Glycemia 11.1 mmol/L and more during OGTT allowed us to confirm DM. If blood glucose level did not reach this level, DM was ruled out, and such animals were withdrawn from further experiments.

If animals developed clinical manifestations typical of absolute insulin insufficiency (polydipsia manifesting in increased water consumption, polyuria, and especially weight loss) which made us suspect type 1 DM, these animals were excluded from further experiments.

Four weeks after DM modeling, the rats were divided into the following groups (n = 10 each):

- “DM”—diabetic animals receiving 0.9% NaCl solution once daily per os;
- “DM+MET”—diabetic animals receiving metformin 200 mg/kg once daily per os for 8 weeks;
- “DM+EMPA”—diabetic animals receiving empagliflozin 2 mg/kg once daily per os for 8 weeks;
- “DM+CANA”—diabetic animals receiving canagliflozin 25 mg/kg once daily per os for 8 weeks;
- “DM+DULA”—diabetic animals receiving dulaglutide 0.12 mg/kg every 72 h s.c. for 8 weeks.

We also took data obtained for the “Control” group (n = 10) in the 1st part of the experiment for comparative analysis.

After 8 weeks, all the animals were subjected to 30 min MCAO, as described above. After 48 h of reperfusion, neurological status was evaluated using Garcia scores. Then, animals were anesthetized and blood samples were collected for NLC, NSE, and S100BB measurement. After euthanasia, brain slices were incubated in 1% triphenyltetrazolium chloride solution for further brain damage volume calculation.

Glycemia was assessed with the help of an AccuCheck Performa (Roche, Basel, Switzerland) glucometer every second week throughout the experiment, since nicotinamide/streptozotocin injection was administered up to MCAO as well as 3 times during the operation of MCAO modeling and in the reperfusion period, no less than three times daily. No measurements were taken after fasting. All animals received an i.p. injection of 1 mL 5% glucose solution at the end of the MCAO operation, 4–5 h after the start of the reperfusion, and thereafter in the reperfusion period if needed.

### 2.2. Statistical Analysis

Statistical data were processed with the help of the IBM SPSS Statistics-22 software package (IBM, Chicago, IL, USA) and Statistica-10 (Statsoft, Tulsa, OK, USA). Nonparametric methods were used. The significance of intergroup differences was measured by nonparametric ANOVA for multiple comparisons (a post hoc pairwise multiple comparison of groups using the Dunn’s test). The results are presented as “median [25%; 75%]”. Significance was reached at *p* < 0.05.

### 2.3. Ethics Approval

The entire experiment was conducted in accordance with the European Convention for the Protection of Vertebrate Animals used for Experimental and other Scientific Purposes and the Guide for the Care and Use of Laboratory Animals (NIH publication No. 85–23, revised 1996). The study was approved by Institutional Animal Care and Use Committee of Almazov National Medical Research Centre (Protocol Number PZ_22_2, 16 February 2022). Every effort was made to protect the animals and minimize distress throughout the study. The experiments complied with the Animal Research: Reporting of In Vivo Experiments (ARRIVE) guidelines (https://arriveguidelines.org/ (accessed on 15 August 2024)).

## 3. Results

### 3.1. I Part: Neuroprotective Properties of SGLT-2i and GLP-1RA in Non-Diabetic Animals

#### 3.1.1. Glycemia Dynamics

None of the study drugs administered to the healthy animals without DM caused hypoglycemia before MCAO modeling. Anesthesia with zoletil + xylazine, as expected, was accompanied by hyperglycemia in the “Control” group—such an effect of xylazine has been described [17,18]. All the study drugs diminished hyperglycemia prominence during the operation, but EMPA, CANA, and DULA were more effective than metformin (MET). In the post-operative period, the glycemic levels in the “EMPA” and “CANA” groups were significantly lower than in the “Control” and the “MET” groups, despite all the animals having received glucose solution at the end of the operation and in the reperfusion period. Nevertheless, no hypoglycemic episodes were noticed in any of the groups (Table 1).

#### 3.1.2. SGLT-2i and GLP-1RA Influence Neurological Deficit and Brain Damage Volume

**Figure 1:**
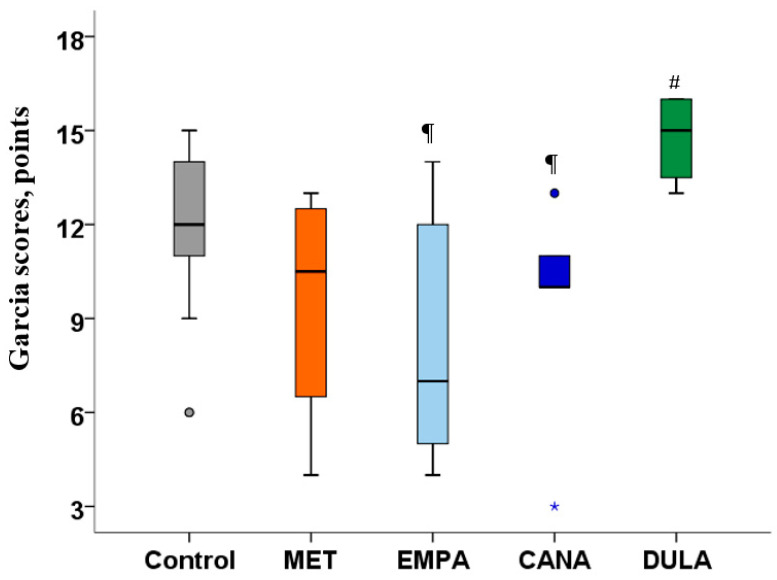
Neurological deficit 48 h after MCAO in non-diabetic rats. # *p* < 0.05 in comparison with the “MET” group, ¶ *p* < 0.05 in comparison with the “DULA” group. MCAO—middle cerebral artery occlusion. Metformin, empagliflozin, and canagliflozin administration for 7 days prior to MCAO did not decrease neurological deficit. Neurological deficit was significantly smaller in the “DULA” group in comparison with all the other treatment groups.

MCAO modeling was accompanied by prominent neurological deficits in the “Control” group. We did not observe improvements in neurological status in either the SGLT-2i or MET treatment groups, while the neurological deficits in the “DULA” group were smaller than in all the other treatment groups (Figure 1).

**Figure 2:**
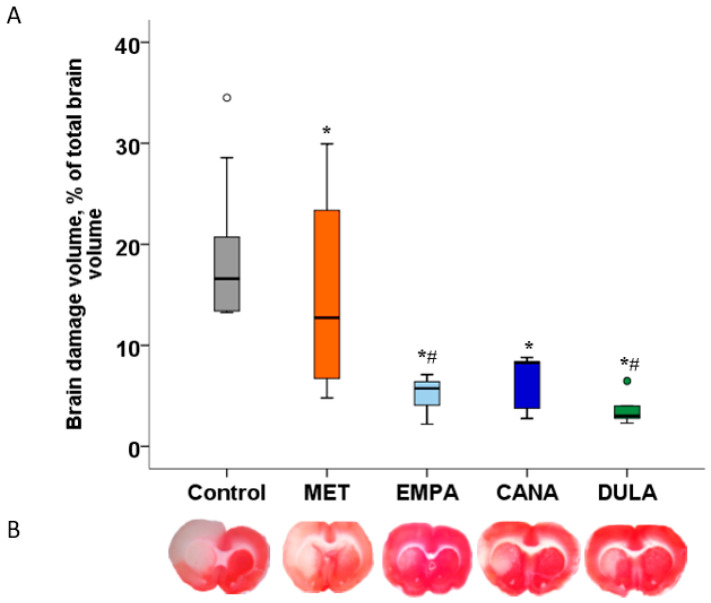
Ischemia–reperfusion injury-induced brain damage in non-diabetic rats. (**A**) Brain damage volume measurement results presented as dot plots with median values. (**B**) Representative images of brain slices stained with triphenyltetrazolium chloride. * *p* < 0.05 in comparison with the “Control” group, # *p* < 0.05 in comparison with the “MET” group. Metformin, empagliflozin, canagliflozin, and dulaglutide reduce brain damage volume in comparison with the control group without treatment 48 h after MCAO in non-diabetic rats. The infarct-limiting effect of empagliflozin and dulaglutide is more prominent than that of metformin.

All study drugs administered for 7 days before MCAO caused significant decreases in brain damage volume in comparison with the “Control” group. The infarct-limiting effect of EMPA and DULA was more prominent than that of MET. No significant differences were observed between both SGLT-2is, nor between SGLT-2i and GLP-1RA DULA (Figure 2).

#### 3.1.3. SGLT-2i and GLP-1RA Influence Concentration of Neuronal and Glial Damage Markers

**Figure 3:**
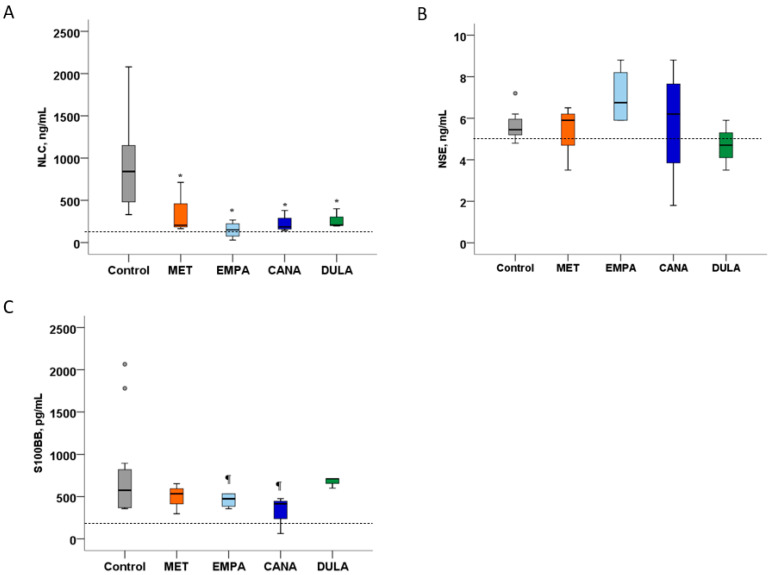
Concentration of neuronal and glial damage markers after MCAO in non-diabetic rats. (**A**) Neurofilament light chains level 48 h after stroke. (**B**) Neuron-specific enolase level 48 h after stroke. (**C**) S100BB level 48 h after stroke. * *p* < 0.05 in comparison with the “Control” group, ¶ *p* < 0.05 in comparison with the “DULA” group. Dashed line—normal value. Ischemic stroke is characterized by NLC, NSE, and S100BB elevation. Metformin, empagliflozin, canagliflozin, and dulaglutide decreased NLC compared with the “Control” group. None of the drugs significantly influenced NSE or S100BB level.

Ischemia–reperfusion injury was characterized by an increased level of NLC in the “Control” group. All the study drugs caused significant decreases in NLC concentration without inter-group differences (Figure 3A). NSE was also slightly increased 48 h after stroke in the “Control” group. Although the median of NSE concentration in the “DULA” group was in the normal range, there were no significant differences either between treatment groups and the “Control” one or among the treatment groups (Figure 3B). The S100BB level was elevated in all the groups. None of the study drugs succeeded in decreasing S100BB in comparison with the “Control” group (Figure 3C).

### 3.2. II Part: Neuroprotective Properties of SGLT-2i and GLP-1RA in Diabetic Animals

#### 3.2.1. Glycemia Dynamics

A high-fat diet and nicotinamide/streptozotocin injection failed to cause DM development in three rats. Therefore, we added three more animals into the experiment in order to maintain the necessary number.

**Figure 4:**
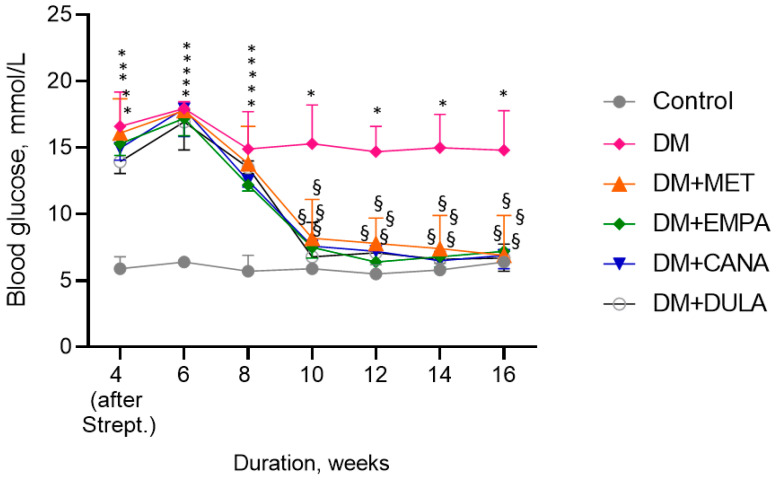
Glycemia dynamics in diabetic rats receiving variable glucose-lowering drugs. Strept.—nicotinamide/streptozotocin administration. * *p* < 0.05 in comparison with the “Control” group, § *p* < 0.05 in comparison with the “DM” group. Eight-week metformin, empagliflozin, canagliflozin, and dulaglutide treatment in diabetic rats caused similar glycemic profile improvement in comparison with untreated diabetes mellitus.

The glycemia dynamics throughout the experiment are shown in Figure 4. Animals in “DM” without treatment group remained hyperglycemic during the entire experiment. Treatment with MET, EMPA, CANA, and DULA led to similar satisfactory glycemic profile development. No differences in glycemia levels were observed among the study groups.

During MCAO, the most prominent hyperglycemia was observed in the “DM” without treatment group. All study drugs caused significant glycemia decrease during the operation, but the effect of MET was less prominent than that of EMPA, CANA, and DULA. There was no significant difference between EMPA, CANA, and DULA in the intraoperative glycemic parameters. We observed normal glycemic values in all the treatment groups in the reperfusion period, with no tendency toward hypoglycemia. The glycemic level in the “DM” without treatment group was expectedly high in the reperfusion period (Table 2).

#### 3.2.2. SGLT-2i and GLP-1RA Influence Neurological Deficit and Brain Damage Volume

**Figure 5:**
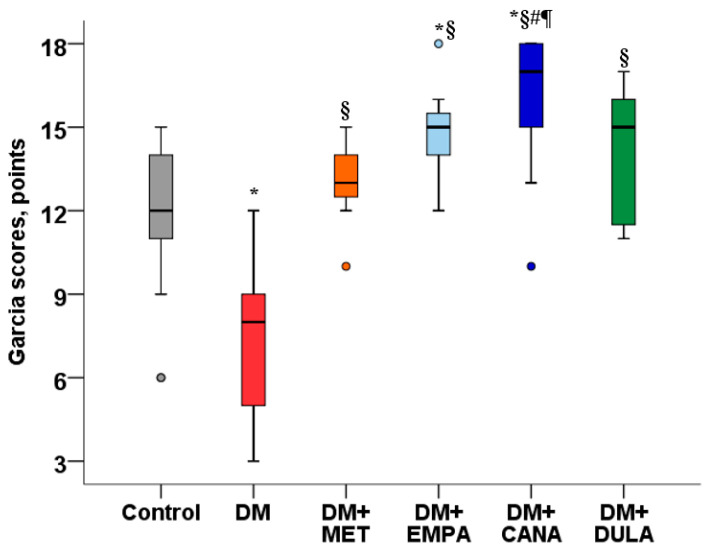
Neurological deficit 48 h after MCAO in diabetic rats. * *p* < 0.05 in comparison with the “Control” group, § *p* < 0.05 in comparison with the “DM” group, # *p* < 0.05 in comparison with the “DM+MET” group, ¶ *p* < 0.05 in comparison with the “DM+DULA” group. MCAO—middle cerebral artery occlusion. Neurological deficit in diabetic rats without treatment was more serious than in the “Control” group. Metformin, empagliflozin, canagliflozin, and dulaglutide improved neurological status. There was no significant difference in empagliflozin and canagliflozin effectiveness, whereas the positive effect of canagliflozin was more prominent than that of metformin and dulaglutide.

Neurological deficit in the “DM” without treatment group was more prominent than in the “Control” group. All study drugs improved the neurological status in comparison with the “DM” group. EMPA and CANA had similar effects on the neurological parameters measured by Garcia scores. The positive effect of CANA was more prominent than that of MET and of DULA (Figure 5).

**Figure 6:**
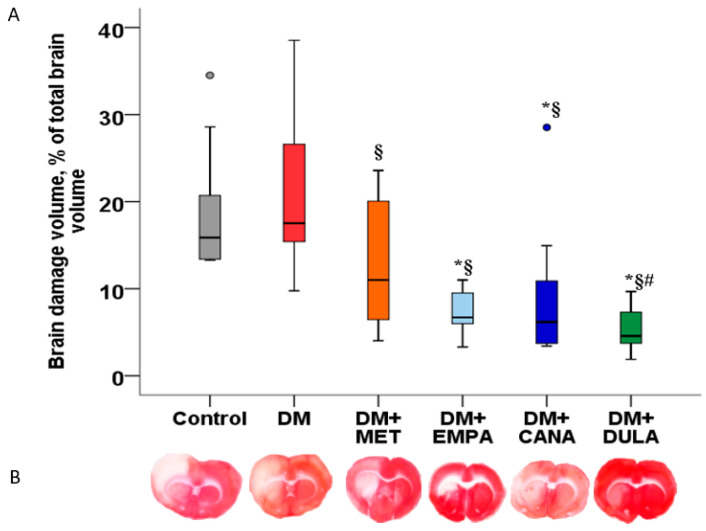
Ischemia–reperfusion injury-induced brain damage in diabetic rats. (**A**) Brain damage volume measurement results, presented as dot plots with median values. (**B**) Representative images of brain slices stained with triphenyltetrazolium chloride. *: *p* < 0.05 in comparison with the “Control” group, §: *p* < 0.05 in comparison with the “DM” group, #: *p* < 0.05 in comparison with the “DM+MET” group. Brain damage volume in the diabetic rats without treatment was as large as that in the “Control” group. All study drugs diminished necrosis volume in comparison with the “DM” group. There was no difference in the infarct-limiting effects of empagliflozin, canagliflozin, and dulaglutide.

The brain damage volume in the “DM” group was as large as that in the “Control” one. All study drugs diminished necrosis volume in comparison with the “DM” group. There was no difference in the infarct-limiting effect of EMPA and CANA, and it did not differ between SGLT-2i and DULA (Figure 6).

#### 3.2.3. SGLT-2i and GLP-1RA Influence Concentration of Neuronal and Glial Damage Markers

**Figure 7:**
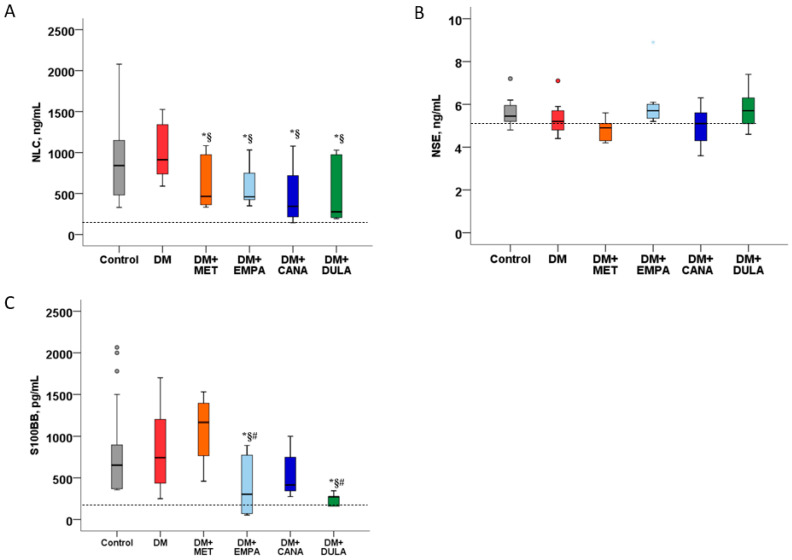
Concentration of neuronal and glial damage markers after MCAO in diabetic rats. (**A**) Neurofilament light chains level 48 h after stroke. (**B**) Neuron-specific enolase level 48 h after stroke. (**C**) S100BB level 48 h after stroke. *: *p* < 0.05 in comparison with the “Control” group, §: *p* < 0.05 in comparison with the “DM” group, #: *p* < 0.05 in comparison with the “DM+MET” group. Dashed line—normal value. NLC concentration was elevated in both the “DM” and the “Control” groups, but metformin, empagliflozin, canagliflozin, and dulaglutide caused similar decreases in it. NSE levels were similar in all the study groups. S100BB was similarly elevated in the “DM” and “Control” groups. Empagliflozin and dulaglutide caused its significant decrease, while metformin and canagliflozin did not influence it.

NLC concentration was elevated in both the “DM” and the “Control” groups. MET, EMPA, CANA, and DULA caused similar decreases in this parameter (Figure 7A). The NSE level was similar in all the study groups, without significant differences among them (Figure 7B). S100BB was similarly elevated in the “DM” and “Control” groups. EMPA and DULA caused its significant decrease, while MET and CANA did not influence it (Figure 7C).

## 4. Discussion

Our study revealed that highly selective and low-selective SGLT-2is and GLP-1RAs are neuroprotective in experimental stroke in both non-diabetic and diabetic conditions. Data concerning the neuroprotective effect of GLP1-RA are rather widely presented. It has been reported that GLP-1RA with different durations of action could reduce brain damage volume and improve neurological status both when used before and after brain ischemia modeling in diabetic and non-diabetic animals [19,20,21,22]. We have also previously investigated and compared the neuroprotective properties of different GLP-1RAs, liraglutide, semaglutide, and dulaglutide, in animals without glucose metabolism impairment and showed that all of them decreased brain necrosis size [10]. As previously mentioned, we chose dulaglutide as the GLP-1RA representative for the current study because its neuroprotective properties have been described not only in pre-clinical trials, but also in clinical ones—dulaglutide is one of two long-acting GLP-1RA that have an opportunity to decrease the risk of non-fatal stroke according to RCTs [6].

Studies on the neuroprotective properties of SGLT-2is in experimental stroke remain limited. There are a few works elucidating the highly selective empagliflozin effect in transient brain ischemia. Thus, it has been shown that empagliflozin administration to diabetic rats in the reperfusion period diminishes brain infarction volume and improves behavioral functions, but in our opinion, the limitation of this study might be connected with the comparative drug gliclazide, the use of which cannot completely exclude the risk of hypoglycemia [23]. Empagliflozin has also been shown to decrease brain damage size in non-diabetic animals when administered before [24] or after transient cerebral ischemia [25]. Some conflicting results were obtained in the study of E. Vercalsteren et al., who showed that empagliflozin improves post-stroke recovery in diabetic animals, but not in non-diabetic ones [26].

Although there are some experimental works focusing on the neuroprotective properties of highly selective SGLT-2is such as empagliflozin, we failed to find studies investigating low-selective SGLT-2is, such as canagliflozin, in transient brain ischemia. Canagliflozin predominantly suppresses sodium-glucose cotransporters (SGLTs) of the 2nd type, which are mostly expressed in the kidneys, but also have an impact on the 1st type of SGLT, which is widely present in different organs, including the kidneys and intestines, as well as tissues not directly involved into carbohydrate metabolism such as the heart and the brain [27]. Although there are limited experimental data concerning the neuroprotective potential of canagliflozin, RCTs have shown canagliflozin’s ability to decrease hemorrhagic stroke risk [28]. Canagliflozin can also improve atrial fibrillation course, thus reducing the incidence of cardioembolic stroke subtype [29]. This neurotropic potential might be partly realized through SGLT 1, as this cotransporter is even more widely expressed in the neurons than SGLT 2 [30].

Taking into account such differences in SGLT-2i pharmacodynamics, we aimed to directly compare the neurotropic action of empagliflozin and canagliflozin as well as to compare their effects with long-acting GLP-1RA. Our preliminary data showed that, in non-diabetic animals, administration of empagliflozin, canagliflozin, and GLP-1RA before stroke has similar infarct-limiting effects [10,15].

Here, we compared the neurotropic effects of EMPA, CANA, and DULA in both non-diabetic and diabetic conditions, as well as attempted to elucidate the underlying mechanisms. We found that EMPA’s infarct-limiting effect does not differ from that of CANA in animals without DM, nor in those with experimental DM. Moreover, the brain damage volume in the EMPA and CANA groups was as small as in the GLP-1RA DULA treatment group independently of the presence of DM. At the same time, both EMPA and CANA, as well as DULA, improved post-stroke recovery in diabetic animals, which was not observed in non-diabetic ones. In the absence of DM, only DULA improved neurological status in comparison with metformin and both SGLT-2is. As has been discussed previously [15], the most reasonable explanation for this phenomenon seems to be the early contra-insulin response down-regulation caused by prolonged SGLT-2i use prior to operation of stroke modeling, combined with post-stroke food deprivation caused by the impossibility of assuming the chow due to prolonged anesthesia-mediated unconsciousness. Although we injected glucose solution during the early reperfusion period in all the study groups in order to avoid hypoglycemia, the glycemia level in the EMPA and CANA groups seemed not to be sufficient for complete functional post-stroke recovery.

In order to investigate the mechanisms underlying the neurotropic effects of SGLT-2i and GLP-1RA, we measured the serum concentrations of neuronal (NLC and NSE) and neuroglial (S100BB) damage markers.

Neurofilaments are members of the intermediate filament family, which consists of cell-specific compounds occupying an intermediate position between actin and myosin filaments by their diameter [31].

NLC comprise a subunit of neurofilaments, which are cylindrical proteins exclusively localized in the neuronal cytoplasm [32]. Neurofilaments are responsible for neurons’ structural stability and are located in dendrites and neuronal soma, as well as in axons, where their expression is especially high. Normally, in physiological conditions, low levels of NLC are constantly released from neuronal axons [33]. However, in response to CNS axonal damage due to inflammatory processes or neurodegenerative or traumatic injury, NLC release is severely enlarged. The serum NLC level correlates with the damage volume in ischemic stroke patients [34].

NSE is a glycolytic enzyme present mostly in the cytoplasm of neurons as well in cells of neuroendocrine origin [35] such as adrenal medulla or thyroid C-cells. It can be released into the blood flow under different pathological conditions involving these tissues, for example, pheochromocytoma or small-cell lung cancer, so it is considered a lung cancer biomarker [36]. Moreover, NSE passes the disrupted blood–brain barrier (BBB) during different variants of brain damage, including traumatic brain injury or stroke. There are some experimental data showing correlations between serum NSE concentrations and the severity of brain damage caused by MCAO [37]. In another study, it has been also shown that NSE is elevated 2 h after MCAO and remains increased for 2.5 days [38].

In the current study, we observed that all glucose-lowering drugs with any infarct-limiting effect influenced NLC levels. Thus, in non-diabetic animals, the NLC concentration in both highly- and low-selective SGLT-2i groups as well as in DULA group was lower than in control untreated animals, without significant inter-group differences. Metformin administration also caused slight NLC, which corresponds to its smaller infarct-limiting effect. We observed a similar tendency in diabetic animals: the NLC level was elevated in untreated diabetic rats following MCAO and both SGLT-2is, and GLP-1RA intensively decreased it. The current study was the first to directly compare the influence of SGLT-2is with different selectivity on neuronal damage markers in both non-diabetic and diabetic animals undergoing brain ischemia, and we can conclude that both highly selective and low-selective SGLT-2is as well as GLP-1RAs realize their neuroprotective properties at least partly through a direct influence on neuronal survival. Nonetheless, we did not observe any differences in NSE between untreated animals or those receiving any kind of treatment in either non-diabetic or in diabetic conditions. This makes us consider that NSE is a neuronal marker that is not sensitive enough to describe the influence of neuroprotective drugs on brain lesions in rat transient cerebral ischemia.

To evaluate the role of neuroglia in stroke and the opportunity of the study drugs to influence it, we measured S100BB serum concentrations. The cerebral S100 family comprises two proteins: S100A1 (S100α) and S100BB (S100β). They are responsible for the regulation of different proteins’ functions and are involved in energy metabolism enzyme activation in brain tissue as well as in cell cycle regulation, the functioning of the cytoskeleton, and the control of cell differentiation and proliferation [39]. S100BB is thought to be specific for astroglial cells. In physiological concentrations, S100BB is essential due to its neurotrophic properties that are realized during neural regeneration or normal development of the CNS. At the same time, when the S100BB concentration exceeds physiological values, there are neurotoxic effects [40]. In physiological conditions, only traces of S100BB can be found in the peripheral blood flow, whereas when BBB is disrupted, S100BB appears in the bloodstream at higher concentrations. Such conditions can include traumatic brain injury, neurodegenerative diseases, or stroke [41]. Moreover, recent studies have indicated that acute brain damage is accompanied not only by S100BB elevation in the bloodstream, but also by such pathologies as type 2 DM and obesity, which are themselves examples of chronic aseptic inflammation [42].

In the current study, we have shown that S100BB is elevated 48 h after MCAO in both non-diabetic and diabetic untreated animals. Thus, it can be considered an indicator of astroglial damage in stroke. Surprisingly, in non-diabetic rats, none of the study drugs succeeded in decreasing S100BB, whereas in diabetic animals, highly selective SGLT-2i empagliflozin and GLP-1RA dulaglutide administration were associated with lower S100B levels. To our knowledge, such differences in the influence of SGLT-2i and GLP-1RA on neuroglia in stroke in diabetic and non-diabetic conditions are being described for the first time.

We suppose that the reasonable explanation for such a phenomenon is that EMPA and DULA modulate prolonged suppression of S100BB accumulation due to DM and obesity. This suggestion corresponds with the knowledge that DM development can be accompanied by microglia activation. Some of the drugs, including EMPA and DULA, while used in diabetic animals for a long period of time, can successfully decrease microglia pathological hyperactivation [14,15]. We can suggest that, similar to the situation described for microglial activation, prolonged use of EMPA and DULA in diabetic rats in our study caused a decrease in astroglial reaction, and therefore, after transient brain ischemia, the S100BB level in the respective groups was lower than in the others. At the same time, in healthy animals without DM and obesity, pathological astroglial reaction before stroke might not take place; thus, there could not be a point of application for prolonged action of SGLT-2i or GLP-1RA before stroke. Their protective properties would need to be realized acutely during the ischemia–reperfusion period and, therefore, predominantly focused on neuron-tropic actions.

Another finding of the current study to be discussed is that EMPA, but not CANA, decreased S100BB levels in diabetic animals. These data correlate with our previous finding that EMPA, but not CANA, can decrease pathological microglia hyperactivation in diabetes without stroke [15]. As mentioned above, EMPA has high affinity to SGLT type 2, while CANA can inhibit SGLT of both the 2nd and 1st type. It still remains under discussion whether SGLT of the 1st and 2nd type are expressed in astrocytes. According to most data, including review ones, neither SGLT 1 nor 2 are expressed in glial cells, only in neurons and nerve cell processes [43]. However, it has been reported that SGLT 2 is expressed in pericytes, and its influence on astrocytes’ carbohydrate metabolism is indirect [44], while SGLT 1 can be expressed in astrocytes themselves, especially in ventromedial hypothalamus at low levels [43,45]. The bioavailability of CANA [46] is lower than that of EMPA [47]. We can suggest that the in-brain concentrations of CANA are not enough to influence neuroglia. On the other hand, CANA demonstrates an outstanding neuroprotective effect in stroke that is realized by means of an alternative mechanism rather than the influence on astroglia that can involve a direct influence on neuronal survival, including the BDNF-mediated mechanism [48].

## 5. Conclusions

We demonstrated that both highly and low-selective SGLT-2is as well as GLP-1RAs are neuroprotective in acute ischemic–reperfusion injury in rats, while in non-diabetic animals, GLP-1RAs are more effective, as they influence both infarction size and neurological status. Stroke development in both non-diabetic and diabetic conditions is accompanied by direct neuronal damage as well as astroglial damage. Both SGLT-2i and GLP-1RA have a direct positive impact on post-stroke neuronal survival independently of DM presence. In the presence of DM, the highly selective SGLT-2i EMPA and long-acting GLP-1RA DULA have an influence on astroglial reaction, while the low-selective SGLT-2i CANA realizes its neuroprotective effect beyond astroglia involvement.

## Figures and Tables

**Table 1:** Glycemia (mmol/L) dynamics in study groups 7 days before, during, and after middle cerebral artery occlusion.

		Control	MET	EMPA	CANA	DULA
Before MCAO	1st measurement	6.8 [5.5; 6.9]	7.3 [5.8; 7.5]	6.6 [5.2; 6.7]	7.1 [6.0; 7.5]	6.7 [6.0; 7.7]
	2nd measurement	6.9 [5.8; 7.2]	7.0 [6.3; 7.7]	6.3 [5.8; 6.6]	6.6 [6.2; 7.2]	6.4 [5.9; 6.8]
	3rd measurement	7.0 [6.5; 7.6]	5.5 [5.0; 6.6]	5.1 [4.5; 6.3]	5.0 [4.3; 6.5]	4.4 [4.2; 6.0]
During MCAO	1st measurement	12.4 [11.7; 14.6]	10.1 [9.9; 14.3] *	9.0 [8.5; 10.1] *#	10.0 [9.5; 10.5] *	8.9 [8.0; 9.5] *#
	2nd measurement	15.5 [14.2; 16.3]	10.5 [9.8; 12.5] *	7.9 [7.0; 9.7] *#	7.0 [6.5; 7.5] *#	7.1 [6.7; 7.5] *#
	3rd measurement	15.8 [12.2; 16.7]	11.2 [10.6; 12.0] *	6.9 [6.5; 10.1] *#	8.0 [7.8; 9.5] *#	7.3 [7.0; 8.0] *#
After MCAO	1st measurement	6.7 [5.3; 6.8]	6.4 [5.5; 6.2]	4.2 [4.0; 5.1] *#	4.3 [4.0; 5.2] *#	6.5 [6,1; 7,1]
	2nd measurement	7.4 [6,8; 8.2]	7.0 [6.7; 7.4]	4.3 [4.0; 5.0] *#	4.5 [4.4; 6,0] *#	6.4 [4.8; 7.2]

Data are presented as median and interquartile range—Me [Q25; Q75]; MCAO: middle cerebral artery occlusion. * *p* < 0.05 in comparison with the “Control” group. # *p* < 0.05 in comparison with the “MET” group.

**Table 2:** Glycemia (mmol/L) dynamics in diabetic rats during and after middle cerebral artery occlusion.

		Control	DM	DM+MET	DM+EMPA	DM+CANA	DM+DULA
During MCAO	1st measurement	12.4 [11.7; 14.6]	22.0 [15.2; 23.5]	15.1 [10.9; 17.3] *§	9.0 [8.5; 10.0] *§	10.0 [9.5; 10.5] *§	8.9 [8.0; 9.5] *§
	2nd measurement	15.5 [14.2; 16.3]	19.6 [18.5; 22.7]	14.5 [11.8; 15.5] *§	7.9 [7.0; 9.7] *§	7.0 [6.5; 7.5] *§	7.1 [6.7; 7.5] *§
	3rd measurement	15.8 [12.2; 16.7]	18.5 [18.0; 20.5]	13.2 [10.6; 14.0] *§	6.9 [6.5; 10.1] *§	8.0 [7.8; 9.5] *§	7.3 [7.0; 8.0] *§
After MCAO	1st measurement	6.7 [5.3; 6.8]	12.7 [12.0; 17.2]	6.4 [5.5; 7.2] §	6.2 [6.0; 7.1] §	5.7 [5.5; 7.2] §	6.7 [6,1; 7,3] §
	2nd measurement	7.4 [6,8; 8.2]	15.8 [15.5; 17.7]	7.0 [6.7; 7.5] §	6.3 [6.0; 7.0] §	6.5 [6.4; 6,8] §	6.4 [4.8; 7.7] §

Data are presented as median and interquartile range—Me [Q25; Q75]; MCAO: middle cerebral artery occlusion. * *p* < 0.05 in comparison with the “Control” group. § *p* < 0.05 in comparison with the “DM” group.

## Data Availability

All datasets on which the conclusions of a manuscript depend are included in the article in the form of graphic material including individual values or boxplots. Additional information is available from the corresponding author upon request.
